# Discovering Composite Lifestyle Biomarkers With Artificial Intelligence From Clinical Studies to Enable Smart eHealth and Digital Therapeutic Services

**DOI:** 10.3389/fdgth.2021.648190

**Published:** 2021-09-06

**Authors:** Sofoklis Kyriazakos, Aristodemos Pnevmatikakis, Alfredo Cesario, Konstantina Kostopoulou, Luca Boldrini, Vincenzo Valentini, Giovanni Scambia

**Affiliations:** ^1^Innovation Sprint Sprl, Brussels, Belgium; ^2^Business Development and Technology, Aarhus University, Herning, Denmark; ^3^Scientific Directorate, Fondazione Policlinico Universitario A. Gemelli Istituto di Ricovero e Cura a Carattere Scientifico, Rome, Italy; ^4^Advanced Radiation Therapy, Fondazione Policlinico Universitario A. Gemelli Istituto di Ricovero e Cura a Carattere Scientifico, Rome, Italy; ^5^Università Cattolica del Sacro Cuore, Rome, Italy

**Keywords:** digital biomarkers, machine learning, ai clinical trials, Healthentia, real world data, e-clinical platform

## Abstract

Discovery of biomarkers is a continuous activity of the research community in the clinical domain that recently shifted its focus toward digital, non-traditional biomarkers that often use physiological, psychological, social, and environmental data to derive an intermediate biomarker. Such biomarkers, by triggering smart services, can be used in a clinical trial framework and eHealth or digital therapeutic services. In this work, we discuss the APACHE trial for determining the quality of life (QoL) of cervical cancer patients and demonstrate how we are discovering a biomarker for this therapeutic area that predicts significant QoL variations. To this extent, we present how real-world data can unfold a big potential for detecting the cervical cancer QoL biomarker and how it can be used for novel treatments. The presented methodology, derived in APACHE, is introduced by Healthentia eClinical solution, and it is beginning to be used in several clinical studies.

## Introduction

The field of clinical research is undergoing a “data revolution.” The transformation of large volumes of medical records to an electronic format, and the remarkable growth in the data collected by health registries and during clinical studies provide opportunities to make risk prediction and intervention selection more precise. This increasing availability of the so-called “Big Data” has brought about a growing interest in machine learning (ML) algorithms for extracting knowledge from observations, typically conceptualized as datasets, and for constructing personalized risk prediction models.

The concepts of real-world data (RWD) and real-world evidence (RWE) have come to be fashionable, along with those that describe outcome and experience from the perspective of the patient [Patient-Reported Outcome Measures (PROMs), Patient-Reported Experience Measures (PREMs)]. The advent of wearable technologies has made the objective measurement of lifestyle possible to an unprecedented scale of dimensionality, and the collection of subjective information about outcome and experience as PROMs and PREMs.

However, in spite of the use of RWD/RWE (and PROMs/PREMs) one important shortcoming in clinical research is that the actual outcome of trials is usually different from the expected one, and often not reproducible (1, 2). Evidence included in the access-to-market dossier of any intervention, pitched to a lesser extent with respect to the expected one, leads to an economic loss in different ways, such as: (i) the tag price agreed upon by regulators at the moment of pricing negotiations drops due to the worse intervention results; (ii) the marketed solution loses competitiveness; and (iii) the overall benefits to the citizens are reduced.

Factors like trial-protocol adherence and compliance, dropout rate, and surveillance of adverse effects have been traditionally outlined as the most significant reasons behind this outcome difference, and several types of interventions aimed at reducing their impact have been put into place (3).

To mitigate unexpected results from clinical trials, along with adherence and compliance issues, digital solutions as clinical diaries have flourished and are vastly used in the running of clinical trials to collect information on how patients are coping through the trial itself by focusing on PROMs. These digital solutions are termed ePRO.

ePROs generally do not take into account the impact of the lifestyle and habits of the patient that can be measured using wearables (the RWD as objectively measured) on the effectiveness of the intervention and focus, instead, on the PROMs and PREMs. In addition to that, lifestyle has not yet been aggregated into actionable predictors, or used to generate simulated models to derive predictors, on outcomes and experiences.

The processing of patient-centered RWD represents an innovative challenge for modern personalized medicine. Today, various patient-well-being dimensions can be satisfactorily met, using merely a multidimensional data collection approach. Data collection platforms, able to collect, manage, and interpret RWD of the patients, eventually supported by artificial intelligence (AI), are fundamental.

In this study, we attempt to establish patient lifestyle and behavior as the driving force of an effective treatment, by addressing the following hypotheses:

1. Objectively-measured RWD correlate to PROMs and PREMs and thus can predict them.
2. The impact of behavior/lifestyle, as expressed by measured RWD, on PROMs and PREMs can be simulated by utilizing biomarkers in models of patients and testing intervention on them.
3. Groups of biomarkers identified *via* simulation of trials lead to behavior/lifestyle phenotypes that can be used as clinical endpoints and eligibility criteria in clinical trials.
4. Coaching on behavior/lifestyle can complete traditional interventions in everyday practice.

This paper is organized around seven sections. Following the paper, after the abstract and introduction, the subjective and objective RWD as clinical outcomes are discussed in section Subjective and Objective RWD as Clinical Outcomes, including definitions and the role of RWD, RWE, and ePROs. section Lifestyle Behavior as a Biomarker With Clinical Value and Types of RWD presents the concept of lifestyle as a biomarker with clinical value, and in section AI Technologies for Defining, Modeling, and Simulating Lifestyle we present how AI can support the discovery and extraction of such biomarkers during clinical studies. Furthermore, in section Pilot Study to Evaluate the Hypothesis, we present elements from a series of clinical studies that utilize the Healthentia eClinical platform (4) to capture insights that can lead to advanced services, and section Expected benefits, Early Findings and Next Steps presents the expected benefits, the early findings, and discusses the next steps. Finally, the conclusions are drawn in section Conclusions.

## Subjective and Objective RWD as Clinical Outcomes

### New Extended Meanings for Old Medical Definitions

A biomarker (5) is the value of a quantity that characterizes the outcome (of a disease) or diagnoses a disease stage. Digital biomarkers (5) are biomarkers whose method of collection involves sensors and computational tools implemented in software or hardware. Traditionally, biomarkers have been split into direct and indirect (6). A direct biomarker is a single measurement of one of the factors or products of the disease that allows diagnosing a disease outcome or stage. Direct biomarkers are usually biochemical, measured obtrusively in a lab. An indirect biomarker is also a single measurement, obtained easily using ubiquitous devices, indirectly associated but highly correlated with a factor or product of the disease. For example, body temperature *T* is an indirect biomarker for flu if:


$$\begin{array}{l} T-37^{◦}C>0\; \end{array}$$

Is body temperature the only biomarker for the flu? No, but it certainly is a good standalone one. Is body mass a biomarker for obesity? Consider a person with a body mass of 120 kg. That person is obese only if their height is <2 m, based on their body mass index (7). Any standalone measurement can give indications on some disease, but the full power of measurements comes into effect when they are combined together into a composite biomarker. A composite biomarker (6) is thus the usually non-linear combination of multiple measurements into a single metric used in disease diagnosis or outcome prediction. In simple cases, the combination can be done analytically, e.g., the body mass index already mentioned is a composite biomarker for obesity that non-linearly combines the height *h* and mass *m* of a person as:


$$\begin{array}{l} \frac{m}{h^{2}}-30>\;0 \end{array}$$

Such simple cases are the exceptions. Usually, there are many measurements forming a vector **x** and the biomarker non-linearly combines them into *F*(**x**). Discovering the non-linear combination function *F*( ) is not done manually, resulting in an equation. Instead, it is done using an ML algorithm that learns *F*( ) for the measurements **x**, yielding the metric to be evaluated for disease diagnosis or outcome prediction. In ML terminology, x is the feature vector, and *F*( ) is the discriminant function of the classifier (8). Hence composite biomarker discovery is about training a classifier or regressor using some ML algorithm.

Finally, a contextualized composite biomarker (6) is the combination of intrinsic factors (that comprise the composite biomarkers) and extrinsic factors, that is, the environment, providing a metric (classifier or predictor output) for personalized disease management.

In sections AI Technologies for Defining, Modeling and Simulating Lifestyle and Pilot Study to Evaluate the Hypothesis of this paper, we are detailing a methodology to discover digital contextualized composite biomarkers in RWD with outcome prediction capabilities.

### RWD and RWE: Definition and Usefulness in Clinical Research

The 21st century Cures Act of 2016, a harbinger of the increasing use of electronic health records (EHRs) and insurance claims data for medical research in the United States, required the Food and Drug Administration (FDA) to develop guidance on the use of RWE in the studies of medical product safety and outcomes for both postapproval studies and studies of new indications of approved drugs. Hence, FDA has issued the following definition: RWD are “*data related to patient health status and/or the delivery of health care routinely collected from EHRs, claims and billing data, data from product and disease registries, patient-generated data including home-use settings, and data gathered from other sources that can inform on health status, such as mobile devices*.” (9). The European Medicine Agency address the same items accordingly, in Organization for Economic Co-Operation and development (OECD)/World health Organization (WHO) (10).

Real-world data are analyzed to create RWE, which is clinical evidence about “*the usage, and potential benefits or risks, of a medical product derived from analysis of RWD*.” (9).

Compared with evidence collected in randomized controlled trials, RWE better reflects the actual clinical environments, in which medical interventions are used, including patient demographics, comorbidities, adherence, and concurrent treatments.

When RWE is intended as the data generated in an observational trial, we notice that there is a significant increase in the number and quality of this type of trial with the consequence of a very significant increase of data assets. It is also well-known that when EHRs are used as a source of RWE, the vast majority of this data, up to 80%, is unstructured. Moreover, insurance claims are a rich source of information and they can cover ontologies not normally included in EHRs, such as the experience of the patients, extensive information on comorbidities (that in EHR is normally highly unstructured), etc., but they are not good for measuring disease severity, biomarkers and, in general, detailed clinical information. In this setting of increasing dimensionality and heterogeneity of data, ML methods are gaining traction as tools to analyze massive and complex datasets (11). When RWE coming from EHRs has been analyzed with ML techniques to create predictive models vs. outcome, these have outperformed traditional ones (12) even in real-time analysis executed, for example, in the emergency setting (13). Some concerns are raised, however, regarding the readiness of EHRs systems to support machine-learning methods from a data quality standpoint (12).

In general, the value of RWE has been well-understood, to the point that many initiatives (and national registries) are amassing insurance claims and EHR curated data.

Real-world evidence derived from observational clinical trials is traditionally collected, objectively as such as data stemming from experimental trials. However, in the observational trials, the setting is entirely uncontrolled and this makes the advancing of “causal learning” to identify direct causes of a certain outcome more difficult. This uncertainty can be overcome by means of modern AI techniques that have proven to be effective, as well, in the setting of synthetic data created by simulation (14). Last but not the least, AI has been proven to mitigate the issue represented by missing data that can impact the process of learning of the causal structure (risk/intervention/outcome). Missing data in real world, moreover, are a significant threat to the understanding of the inference and they are very common; however, AI techniques (in particular Bayesian Networks) do not need complete information on any single record (case/patient) to derive a response variable (15).

To the best of our knowledge, RWD stemming from lifestyle have never been used to train AI-powered simulators alone or along with RWE.

### Patient Reported Outomes: Definition and Usefulness in Clinical Research

The FDA deffinition of Patient Reported Outomes (PROs) is “*any report of the status of the patient's health condition that comes directly from the patient, without interpretation of the patient's response by a clinician or anyone else*” (16). Indeed, PROs may be referred to symptoms related to a disease, functional statuses or multidimensional constructs such as, for example, the health-related quality of life, as defined in Revicki et al. (17).

PROs are currently used as clinical trial endpoints following a constant increase of their recognition as such over the last two decades. Along with PROs, other measurements related to the patient–reported experience and patient-reported behavior are being used more and more as endpoints in clinical trials.

Structured and validated PROs reduce significantly the heterogeneity of the responses of patients making possible, to a higher extent, the understanding of the real differences in the perception of the outcome, as compared with the information collected *via* open-end questions. In this setting, the value of PROs is not only recognized by the regulator and competent authorities but, as well, by many scientific societies.

PROs can be used as primary, secondary, or even exploratory/tertiary endpoints for the hypothesis generation. Interventional trials experimenting with a new medicinal product do have PROs, normally, as secondary endpoints, whereas palliative care trials or rehabilitation ones can have PROs as primary endpoints.

Following the lines of simplification and according to (18), the benefits of including PROs into clinical trials are:

1. Better understanding of the cost/benefits of a treatment;
2. Better understanding of the patients' experience beyond the biomedical outcomes, especially in the domains of pain, fatigue and inconvenience from any other symptom;
3. Better tools to improve methodologies of trials.

From the regulatory perspectives, the strength of the methodology PROs used in the trial could allow the achievement of the status of “PRO labeling” for approved products, which, in turn, allows PRO-supported claims.

We consider PROs, thus defined and characterized as very reliable endpoints, to measure, in conjunction with the impact on the PROs themselves, the variations in the lifestyle behaviors; these are discussed in the following paragraph under the assumption that they may be considered as biomarkers with clinical value.

## Lifestyle Behavior as a Biomarker With Clinical Value and Types of RWD

Lifestyle behavior includes all features that characterize the daily living of people, without considering possible diseases in a direct manner. The importance of observing lifestyle behavior lies upon the evidence that lifestyle is a health determinant and has a two-way link with the disease. A patient with a chronic disease can see the health deterioration mapped into their lifestyle behavior, whereas changes in daily living can have a significant contribution to health, besides any intervention provided.

### Lifestyle and Health

There are several studies that provide solid evidence about the relation of lifestyle with health. A study related to diabetes prevention (18) suggests that lifestyle behaviors are important to the outcomes in youth and adults, with evidence that obesity in adults has risen from <5% to more than 40% in some states, and similar increases in prevalence have been seen in type 2 diabetes, a disease that has increased in prevalence over the last 20–30 years.

Another study (19) shows that good-health-promoting lifestyle behavior, especially health responsibility, physical activity, and stress management behavior are determinants of overweight and obesity, which are major risk factors for the development of cardiovascular diseases, type II diabetes, and some form of cancer.

We quantify lifestyle by obtaining RWD in four fields: physiological, psychological, social, and environmental. Information in all these fields can be objectively measured using devices, or can be subjectively reported by people by answering questionnaires. This information will form the constituents of our proprietary composite biomarker, making up the feature vector to be used as an input to the underlying ML algorithm implementing this biomarker. Not everything we are discussing in the following subsections will be used in the final biomarker. As it is presented in section Composite Lifestyle Biomarker Discovery, domain-knowledge, and feature-importance analysis of the biomarker design process will drive the selection on a per-case basis, but here we give the extensive list.

### Types of RWD

We have identified several types of RWD that can be grouped into four categories. In the physiological category of RWD, we mainly encounter RWD that are mostly measured using activity trackers and/or smartphones. Activity-related features are steps walked, distance, elevation, energy dissipation, time spent in different activity intensity zones (e.g., mild, moderate, and high intensity physical activity, as it is formally defined as a function of age) and exercise activities (walking, running, cycling, etc.), and their distribution in the day. Presence indoors or outdoors is also of importance. Specialized physical activity is also measured *via* composite tests like the 6-min walk, the frailty test, or games specifically designed to measure muscular responses (taping on a mobile phone screen for Parkinson's disease or performing other exercises which are monitored and analyzed by depth cameras to measure features important in stroke or accident rehabilitation). All these tests are scripted and hence can be measured using sensors and audiovisual instructions to the people on their smartphones. Heart-related features include the continuous measurements of the heart rate variability, the time spent in different heart rate zones, and the daily resting heart rate measurement. Sleep-related features include continuous measurements on the time spent in the different sleep stages (awake in bed, light, REM, deep sleep). More physiological aspects are reported. We collected reported symptoms (e.g., headache, body temperature, blood pressure, pains, diarrhea, fatigue, nausea), including their intensity. We regularly collected reported weight and height also. Nutrition is paramount, starting at a higher level with the number of meals in the day and the main ingredient of the meal (plant vs. meat-based meal), but a more detailed analysis can also be used when available. Water, coffee, and alcohol intake are reported, and so are toilet visits. Finally, the menstrual cycle is also of importance.

Most RWD types in the psychological category are reported and include a high-level simple emotional state self-assessment or the 11 aspects of the OECD better life index (20), but when deemed necessary the collected information goes deeper using standardized reports from professional therapists who are monitoring the patients. Measurements can also be used to indirectly capture psychological aspects. Emotion can be recognized from the face video, the voice audio, or the social media text posts. Places visited (which, how diverse they are) are also an indication of the psychological state. Aspects like the weather or spending unusual time in commuting can have some importance.

Real-world data in the social category can be measured indirectly from the usage of the phone (diversity, duration, and frequency of calls) and social media (diversity, number, and frequency of interactions). More direct information can be reported using questionnaires on activities with friends, family, or co-workers.

Real-world data in the *environmental category* include environmental indicators for the assessment of the quality of life (*QoL*) that can be reported by the patients using questionnaires. Precise measurements of living or working environment quality can be obtained by integrating relevant commercial devices (e.g., for air quality analysis).

## AI Technologies for Defining, Modeling, and Simulating Lifestyle

Risk and outcome predictions in clinical medicine have become more precise due to the remarkable growth in the data collected, and with RWE and the growing interest in AI techniques, the construction of personalized risk and outcome prediction models is now more robust.

### Composite Lifestyle Biomarker Discovery

Our digital biomarkers are composite contextual ones, in the sense, that they comprise numerous diverse (usually) indirect measurements, including environmental aspects (5). In Guthrie et al. (21) a methodology for discovering digital biomarkers is introduced that comprises choices on outcomes, features and modeling techniques, and model validation and explanation. Our biomarker discovery is a variation of this methodology. We propose a workflow of specifically designed trials where our biomarker discovery is done in three stages (definition, RWD selection, and iterative design), followed by performance assessment. Our contribution is the introduction of the iterative design where we follow the steps of Guthrie et al. (21) in validating and explaining the models, but we also use the results of this explanation to redefine our RWD selection and retrain the model in iterations.

During the *biomarker definition stage*, the domain experts select the clinically significant outcomes that need to be predicted by the biomarker(s). In most cases, the investigators are interested in whether these outcomes are reached or not by the patients. Then the biomarker is implemented as a binary classifier that predicts success or failure in reaching the outcome (21). There can also be cases where it is interesting to predict the actual values of the different outcome quantities. Then, either a classifier with discrete states predicts outcome value ranges (21), or a regressor predicts an outcome value (22). In essence, this definition stage leads to the number of biomarkers needed, and the underlying ML algorithm family (predictor—binary or multiclass, or regressor) to be employed for implementing each of them.

Other aspects that have to do with training the ML algorithms are also defined at this stage: Primarily, based on the different algorithmic needs and the clinical considerations, the ideal amount of data that needs to be collected is established by deciding on the number of trial people participants and the duration of the trial. If the biomarker is expected to be used during the trial, then the training period of the biomarker needs to be defined. During this training period information is collected to train the ML algorithm but no prediction is attempted. If the purpose of the trial is to discover the biomarker for future use, then the split of the trial population into training, validation, and testing datasets is defined. The design stage is carried out prior to the trials, and the choices made are reflected in their protocols.

In the *biomarker RWD selection stage*, domain knowledge is applied to manually narrow down the list of RWD in all four fields discussed in section Lifestyle Behavior as a Biomarker With Clinical Value and Types of RWD, into those that are relevant to the disease/condition in question. Only established irrelevant RWD are omitted at this stage, since one purpose of the biomarker discovery is to establish if some aspects of RWD discussed in section Lifestyle Behavior as a Biomarker With Clinical Value and Types of RWD do impact the condition in question. Another factor for the RWD selection is the ease of measurement. RWD that are collected unobtrusively are usually in the initial selection, since its collection does not impact the everyday life of the participant. RWD requiring manual input using complicated questionnaires needs to be adequately justified. A user-centric design of the interface of the ePRO greatly helps at this stage, since it can remove the burden of collecting certain RWD. The outcome of this stage is the identification of the initial constituents of the feature vectors to be used as input to the ML algorithms implementing the biomarkers. This stage is also carried out prior to the trials to finalize their protocols.

The core of our biomarker discovery workflow is the biomarker iterative design stage. It involves the iterative retraining of the classifier or regressor implementing the biomarker. In this loop, the ML algorithm is used to train the biomarker using the current version of the feature vector. After training, the results are analyzed to refine the feature vector and repeat the process as long as the validation results are improved. The initial training happens when the first outcomes are collected after the end of the training period. Such outcomes can be intermediate ones, or even the final ones, meaning that the biomarker discovery cannot enter phase three before the end of the trial. At the end of the process, the feature vectors of all people are collected, together with the actual outcomes for the duration of the trial. During the iterations of this design stage, the training set is used to train the ML algorithm of choice. The choice depends on the problem at hand, the most determining factors being the size of the training set and the dimensionality of the feature vector. Classifiers that are able to uncover nonlinear decision surfaces are preferred, namely subclass linear methods (23–25), Kernel methods (26), random forests (27), and (deep) neural networks (28). The validation set is used in iterations to tune the parameters of the underlying ML algorithm of the biomarker and determine which of the feature vector elements strongly contribute to the predictions of the biomarker (either toward positive or negative outcomes). Such an analysis can be done using Pearson correlation coefficients (29, 30) or, more importantly, Shapley additive explanations (SHAP) analysis (31–33). SHAP analysis is applied on every feature vector instance, yielding the effect of each feature vector element toward a positive or negative prediction. *Via* SHAP analysis, we identify those feature vector elements that are consistently not contributing to either positive or negative predictions, and those feature vector elements whose value groups (large, medium, or small) do not consistently push toward a positive or negative prediction.

**Figure 1:**
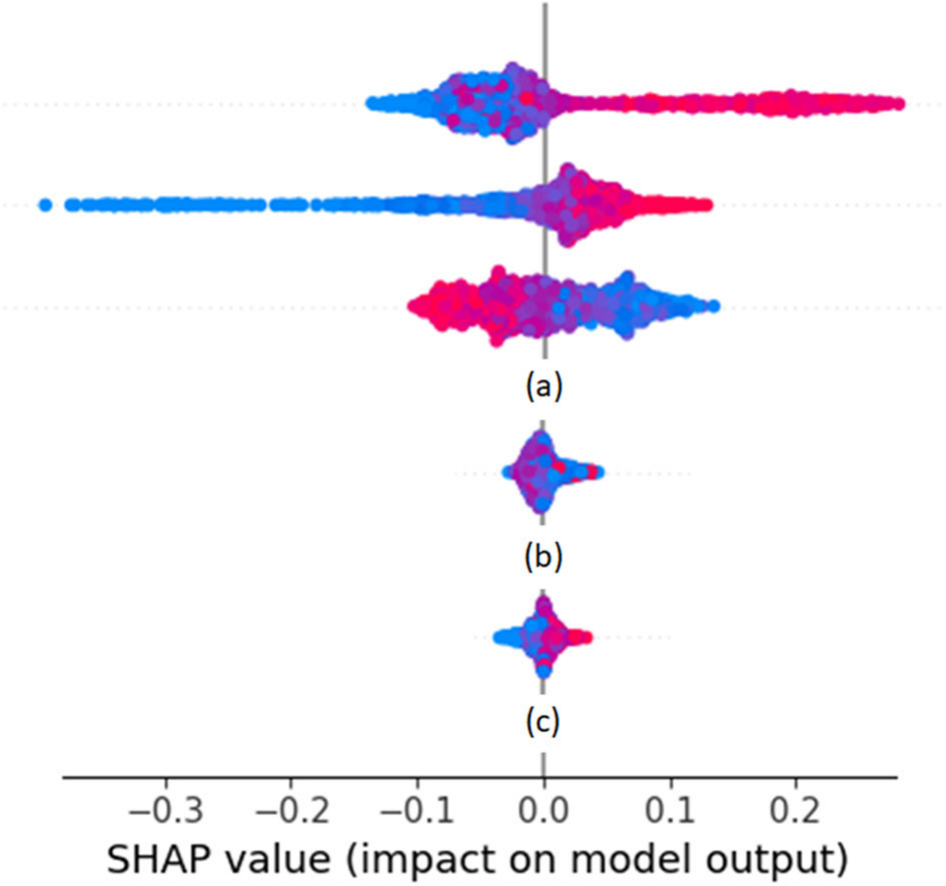
Example SHAP values from a Random Forest classifier predicting weekly health variation.

Cases of SHAP values are shown as rows of point clouds in Figure 1. Each point cloud (row) corresponds to a feature vector element, whose importance in the overall decision is being assessed. Each point in the clouds corresponds to the corresponding element of one feature vector on which the decision is based. The color of the point indicates the value of the element (from small values in blue to large values in red). The placement of the point on the horizontal axis corresponds to the SHAP value. Values close to zero correspond to feature vector elements with negligible effect on the decision, whereas large positive or negative values correspond to feature vector elements with large effect. The vertical displacement of the point within its row indicates how many feature vectors fall into the particular range of SHAP values. Thus, thick point cloud areas correspond to many feature vectors. The point clouds marked as (a) correspond to feature vector elements that have a large impact on decisions (either positive or negative). The point cloud marked as (b) corresponds to a feature vector element whose large, medium, or small value seems to push the decision to random directions. The point cloud marked as (c) corresponds to a feature vector element that has a small impact on decisions. Feature vector elements falling in any of the categories marked as (b) or (c) are candidates to be dropped in the next iteration of biomarker retraining.

**Figure 2:**
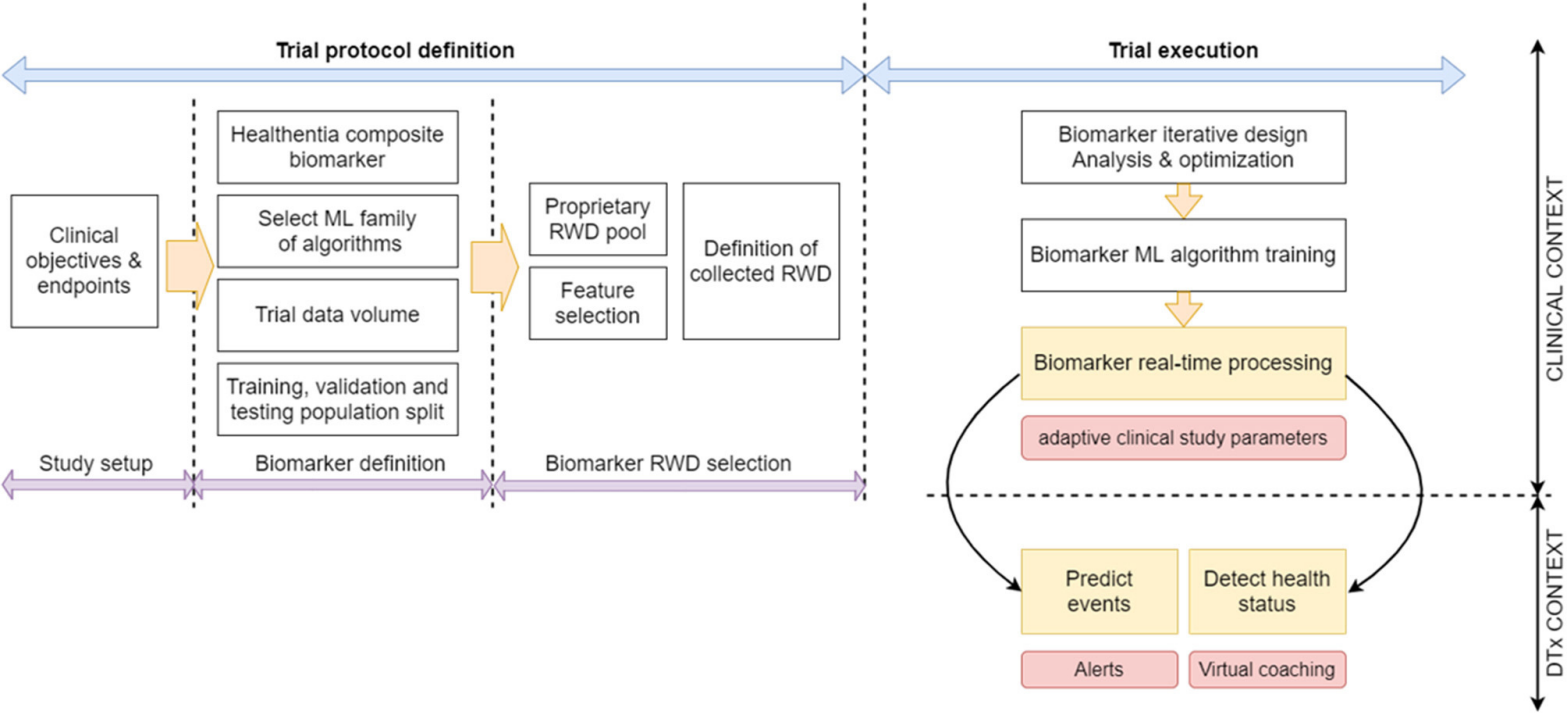
Proposed biomarker discovery and application trial workflow.

The performance of the biomarker is assessed at the biomarker performance assessment stage that follows after the iterative process. The RWD test set is used at this stage. It needs to be noted that for biomarkers predicting final trial outcomes, there are only as many feature vectors as there are patients in the trial. When the number of participants is low, then the validation stage is done using the test set itself, or in more tight cases the process is run repeatedly employing the “leave one out for testing” method, where all features are individually used to test classifiers or estimators trained and tuned using all the rest. The three biomarker-discovery phases and the resulting biomarker assessment are summarized in our proposed workflow for biomarker discovery trials, shown in Figure 2.

### Using the Biomarkers for Digital Therapeutics

By employing AI discovered biomarkers that successfully predict clinically significant outcomes, it is possible to drive decisions in digital therapeutics (DTx) (21, 34, 35). When a disease is considered, then the aim is to drive the intervention. The biomarker predictions indicate intervention strength (drug dosage), which usually is not one of the feature vector elements. The usage of a biomarker in DTx involves balancing the trade-off between its specificity (its ability to correctly identify those patients who do not achieve the desired clinical outcome) and its sensitivity (the ability to correctly identify those patients that achieve the desired clinical outcome). Usually, high specificity is required not to reduce the intervention to patients who actually need it. If at that high specificity the biomarker also yields high sensitivity, that is, identifies the patients who should receive less strong intervention, then the biomarker is a successful one. This is usually quantified by the area under the receiver operating characteristic (ROC) curve.

Digital therapeutics is also applied in the more general, disease agnostic, and well-being areas. In this area, a biomarker is used to drive behavioral change in a virtual coaching setup. The explainable AI elements already discussed in the previous section determine the elements of the feature vector of the particular patient who had the most positive and negative influence on the probability of a successful outcome. Then the person is coached in these elements. The virtual coach selects the feature vector elements of the strong influence that are related to the behavior (physiological, psychological, and social aspects) and to the environment. If they have a strong negative influence, it coaches the person to change behavior. If they have a strong positive influence, it encourages the person to keep up the good lifestyle in those aspects.

While recently the use of ePRO and digital monitoring devices in clinical trials is ever-increasing, and many of those aim at deriving digital biomarkers (6), to the 'knowledge of the author there are no trials that already have evaluated DTx applications. It is our hypothesis that our digital biomarkers and the SHAP analysis of their individual decisions can be applied in a DTx context by driving coaching of the patients.

## Pilot Study to Evaluate the Hypothesis

### Study Description

The study APACHE, which is an advanced patient monitoring and AI-supported outcomes assessment in cervical cancer using Internet of things technologies, is a cofinanced monocentric observational study using a remote monitoring device for patients affected by locally advanced cervical cancer. Patients are considered as such if staged larger than or equal to IB2 according to the FIGO staging system (an international staging system for locally advanced cervical cancer), with primary lesions larger than 4 cm. The patients undergo chemoradiotherapy (CRT) followed by either radical surgery or brachytherapy boost and are treated in Fondazione Policlinico Universitario “A. Gemelli” IRCCS of Rome, Italy. The foreseen study duration is 24 months. The study protocol foresees inclusion and exclusion criteria. The inclusion criteria require the patients to be younger than 70 years, be clinically able to use portable technologies, and be able to understand and sign informed consent. The exclusion criteria involve a major psychiatric disorder, inadequate performance status (larger than 3 according to the Eastern Cooperative Oncology Group score, that is, capable of only limited selfcare; confined to bed or chair for more than 50% of waking hours), and ongoing pregnancy or breastfeeding. Patient enrolment began in October 2020 and continues to date. A total of 50 patients are foreseen for this exploratory study. The selection procedure of patients adhering to the study protocol criteria foresees only a brief interview during the first visit to the advanced radiation therapy center of Gemelli for the initial radiotherapy treatment. During the interview, the informed consent is acquired by the attending physician, and papers describing the trial and expected role of the patient are provided. If the patient is motivated and computer literate, the whole procedure does not take more than 15–20 min. Please note that the initial inclusion criteria on cervical cancer has been widened to include other pelvic cancers, as discussed in section Expected Benefits, Early Findings, and Next Steps.

The primary objective of the study is to assess the experience of patients using Healthentia (see section Healthentia Platform) and a wearable tracker during the multimodal oncological therapies and follow-up period. The study has three secondary objectives. Firstly, to compare PROs with corresponding clinical records about toxicity, instrumental activities of daily living (IADLs), and stress/coping levels. Secondly, to profile patients based on their scores and activity, and lastly, to train models using ML on the patient-reported and monitored data.

Patients have received a state-of -the-art wearable device (Fitbit INSPIRE) during their first visit prior to CRT start, that collects at a daily basis RWD like activity (i.e., steps per day), sleep, and vital signs. During the whole observation period, patients are asked to report their weekly well-being by completing dedicated questionnaires sent to the ePRO App on their phone. A dedicated research nurse will flank the patients enrolled in the study in filling the e-questionnaires in case of need and follow up the correct flow of the questionnaires.

The following scoring systems are selected to assess specific aspects of the experience of the patient during the multimodal treatment period:

1. Early and late toxicity will be assessed using the NCI-PRO-CTCAE™ ITEMS-ITALIAN Version 1.0 for the cutaneous, gastro-intestinal, and genito-urinary sections
2. Therapy impact on instrumental daily activities will be assessed using the Lawton Brody IADL
3. QoL will be assessed using the EORTC QLQ C30
4. Nutritional status will be assessed using the malnutrition screening tool
5. Psychological status will be assessed through self-administered tests, namely the distress thermometer, DT6 for distress evaluation and the Mental Adjustment to Cancer Scale, MINI-MAC 7, Italian version for coping evaluation
6. User experience and technology acceptance will be assessed using a customized questionnaire on the Healthentia App on enjoyment, aesthetics, control, trust in technology, perceived usefulness, and intention to use.

The collected data are transferred through the Healthentia app on the smartphone of the patient to Healthentia platform. Clinicians can monitor lifestyle behavioral patterns, detect changes in the health status, and be informed on clinical endpoints *via* the Healthentia portal web application. More information on the Healthentia eClinical solution is given in section Healthentia Platform.

The collected RWD, i.e., objective data from wearable devices and subjective data collected from questionnaires (e.g., IADLs, toxicity, QLQ, etc.) are fused together and define a multidimensional vector for each patient that consists of steps, resting heart-rate, sleep, etc., which characterizes their behavior throughout the day. After the models are trained from the RWD collected over an initial period, it is possible to predict outcomes and system scoring of the above scoring systems i.e., IADLs or QLQ, by feeding the system with the automatically collected vectors.

The RWD that is being collected is currently grouped into five lifestyle aspects described in Table 1. Most of the aspects can be considered generic (applicable to people in general, not just cervical cancer patients). Only the aspect on QoL, since it focusses on low toxicity adverse effects, is dedicated to the particular condition under study in APACHE. Each aspect is measured *via* a set of parameters (different measurements of questions). The parameters are concatenated into a set of scores, as described in the section on the manual RWD selection stage. It is through these scores that the five aspects are quantified.

**Table 1:** Lifestyle aspects grouping of the Real-World Data collected in APACHE, their parameters, and their scores.

**Lifestyle aspect**	**Questionnaire/measurement**	**Parameters/score**
Physiological	Fitbit activity measurements	16 parameters, aggregated into 4 scores: sleep quality, steps, activity score, resting heart rate
Independence	Instrumental Activities of Daily Life (IADL)	8 parameters, aggregated into 1 score
QoL (Low toxicity adverse effects)	EORTC QLQ (CX24, CX30)	25 parameters aggregated into 4 scores
	NCI/DRO/CTCAE	45 parameters, aggregated into 5 scores
Psychological	Mini Mac Scale	40 parameters, aggregated into 5 scores
	Distress thermometer	1 parameter/score
Nutrition	Nutrition score	1 parameter/score

### Manual RWD Selection Stage

Each of the five lifestyle aspects (see Table 1) combines multiple parameters (measurements or questionnaire answers). In most cases, these parameters are aggregated into one or more scores quantifying the performance in the respective lifestyle aspect. The physiological lifestyle aspect parameters are measured on a daily basis using Fitbit activity trackers. Four scores are derived from these measurements. A sleep score is derived from the total sleep duration, and the REM and deep sleep durations, the sleep efficiency (ratio of time being asleep, over the total time in bed), sleep disturbances (count of wake-up times), and the bedtime alignment to the habits of the patient. An activity score is determined by the active vs. inactive time (excluding sleep), positive or negative deviation from habits, and auto-detected training count. Steps are used as a standalone score since they are the most usual activity tracking metric and are easy to compare against different activity trackers. Finally, the resting heart rate is another standalone score due to its clinical importance as a biomarker on body condition.

The independent lifestyle aspect is reported on a weekly basis using the IADL questionnaire. There are eight questions covering telephone use, shopping, food preparation, housekeeping, laundry, transportation, medication adherence, and finances management. Each of these parameters contributes equally to the overall independence score.

The QoL lifestyle aspect is assessed using two questionnaires. The EORTC QLQ assesses parameters on symptom experience (15 parameters, assessed weekly), body image (three parameters, assessed weekly), sexual/vaginal functioning (four parameters, assessed weekly), and sexual worry/activity/enjoyment (three parameters, assessed monthly). These four groups are the four scores from EORTC QLQ, three of them obtained weekly and one monthly.

The NCI/DRO/CTCAE questionnaire collects 45 parameters on a weekly basis, all having to do with different symptoms (their occurrence, frequency, and/or distress level). They are grouped into five categories, the gastrointestinal (16 parameters), the skin (13 parameters), the neuro (2 parameters), the sexual (2 parameters), and the urinary (8 parameters). There are also two parameters that cannot be classified in the above groups of interest and are ignored by our scoring of the APACHE outcomes. They have not been removed from the data collected to maintain the integrity of the questionnaire used. These five categories are the five scores from NCI/DRO/CTCAE, obtained weekly.

The psychological lifestyle aspect is also assessed using two questionnaires. The Mini Mac Scale assesses (every 3 months) parameters that are aggregated into scores (all parameters contributing equally to their respective scores) on the fighting spirit (16 parameters), helplessness/hopelessness (six parameters), anxious preoccupation (nine parameters), fatalism (eight parameters), and denial/avoidance (one parameter).

The distress thermometer comprises a single parameter forming a score on a weekly basis. Finally, the nutrition lifestyle aspect comprises a single parameter on malnutrition score collected on a weekly basis.

Summarizing, the biomarkers discovered in APACHE utilize 66 parameters and/or their grouping into 12 scores as a feature vector. They are being trained to predict significant variations in the three scores quantifying QoL from the EORTC QLQ questionnaire. Training is done on anonymized APACHE data using proprietary scripts built on top of well-established implementations of ML algorithms found in the Scikit Learn and Tensorflow libraries.

### Healthentia Platform

**Figure 3:**
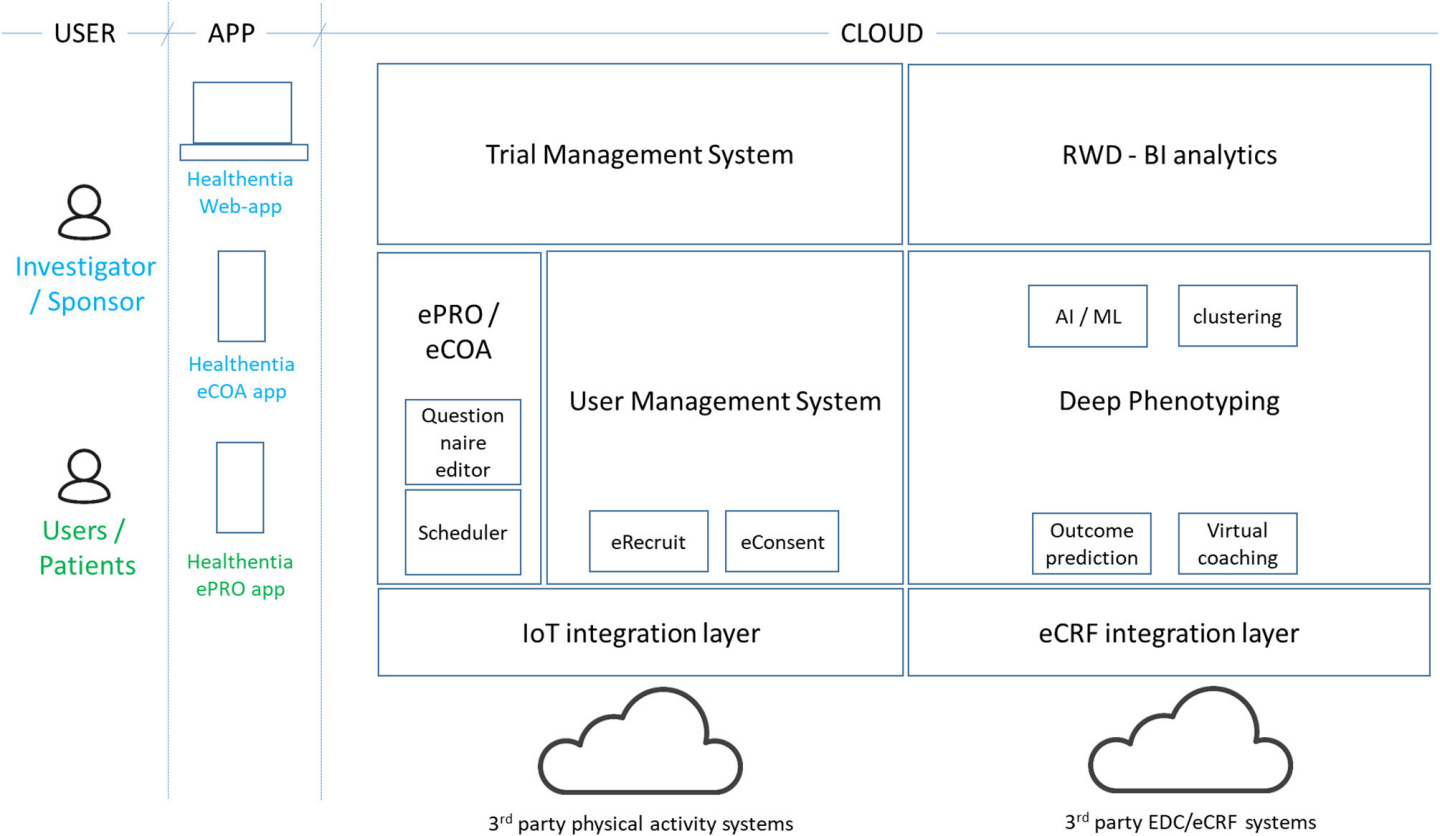
Healthentia architecture.

Healthentia (4) is an eClinical solution that facilitates clinical trial optimization by accelerating the trial processes, reducing the failure rate, and validating drug/intervention efficacy and effectiveness with RWD insights. In this way, pharmaceutical companies can achieve cost savings, accelerate the drug approval process, and obtain useful insights to develop drugs and interventions of higher efficacy. Its architecture is shown in Figure 3.

The Healthentia solution extends the use of a traditional ePRO/eCOA application by adding behavioral and health-related data collected from Internet of Things (IoT) devices. Utilizing ML on this data, it is possible to discover behavioral biomarkers and cluster patients into behavioral phenotypes, which allows the activation of smart services to predict clinical outcomes, generate prevention alarms, and link phenotypes with drug/intervention efficacy. In addition, based on reported outcomes, the AI module generates automatic alerts in case of adverse events. These automatic and prevention alarms support decision making by the investigator during the clinical trial, for the benefit of the health of the individual patient. For the AI module to do so online, the biomarker needs to be trained first as discussed in section Manual RWD Selection Stage.

On top of *in vivo* clinical studies, Healthentia allows the running of *in silico* trials that use the deep phenotyping outcomes together with legacy data, to create synthetic control arms and support pharmaceutical companies to design optimized studies.

Healthentia is available for clinical studies, under a strict regulatory framework, and a SaaS environment, which is open to the wider community. The SaaS version includes further features, such as eRecruitment, eConsent, and Virtual Coaching. Healthentia is already in use in APACHE, and we have received ethical clearance for its use in more studies with a top pharmaceutical company and a hospital. Results from its AI module (for biomarker training) have been published (36), albeit in a completely different domain.

## Expected Benefits, Early Findings, and Next Steps

**Figure 4:**
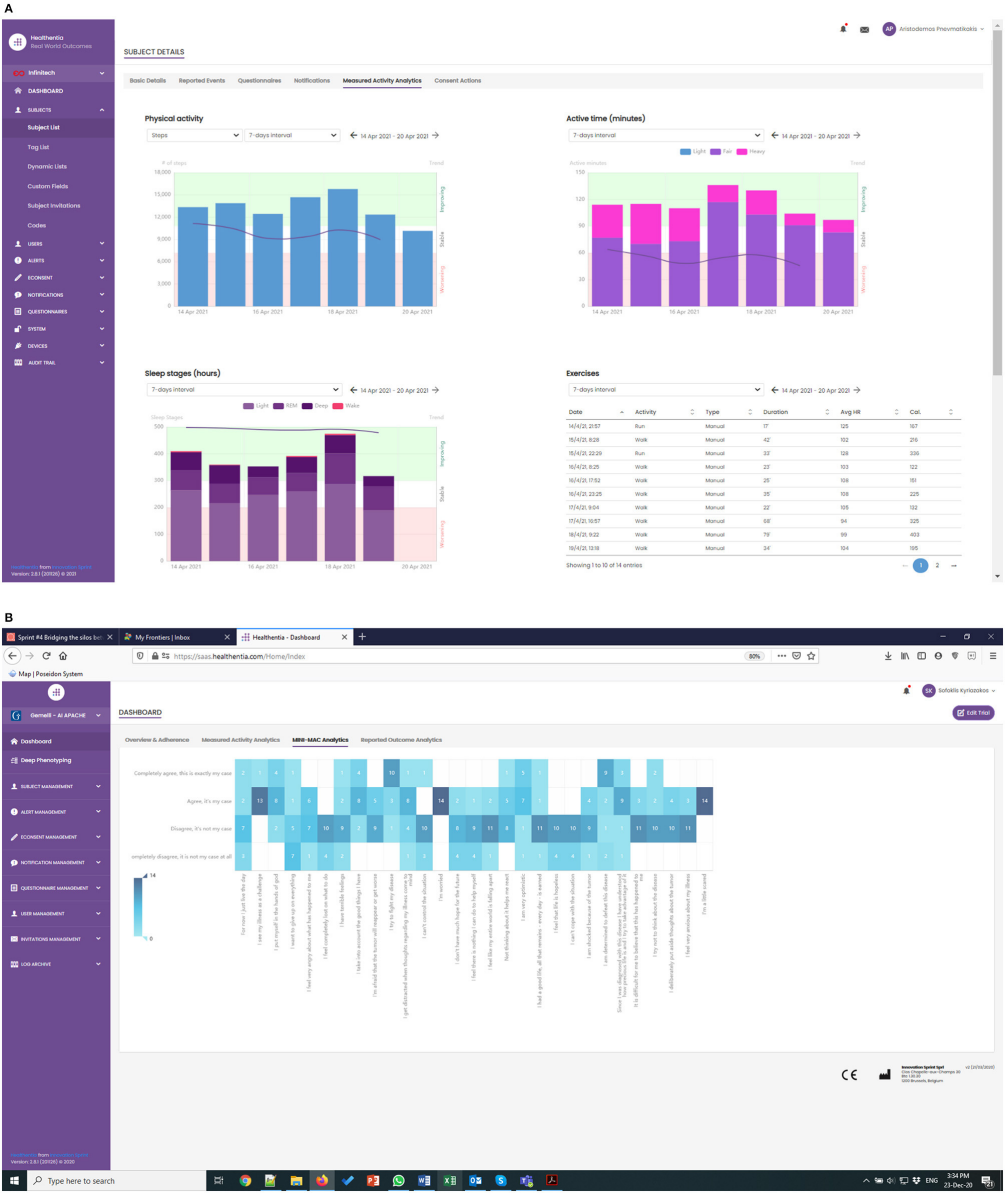
**(A)** Monitoring of physical activity, and **(B)** low toxicity events in APACHE trial.

The APACHE study has been running for a few weeks now, albeit with lower enrolment rates than expected. The first RWD are being collected, as shown in Figure 4, where the real-time monitoring functionalities of Healthentia offer investigators views of the RWD, like the depicted physical activity and MINI-MAC cancer scale.

In the APACHE study, we have introduced new patient-centered variables for risk stratification (i.e., toxicity onset, malnutrition, or mental coping issues), allowing the prospective setup of rapid and fully personalized therapeutic approaches. The integration of such variables into clinical nomograms and multidimensional predictive models is contributing to realizing decisional support systems. The study contributes to the active monitoring of toxicity and therapy-related side effects, aiming at their reduction, and in the optimization of monitoring and follow-up strategies of the patients. Finally, the use of Healthentia enhances self-awareness of the patients about global clinical status and participated clinical decision making.

Clinicians expect different benefits from the use of such advanced monitoring techniques. First of all, the reduction of toxicities may hamper QoL of the patient and their compliance to the oncological multimodal treatments, thanks to the identification of alert signs and early symptoms that may be overlooked during the visits or considered negligible by the patients themselves and not properly reported to the attending physician. This may be translated into an active personalization of the ongoing treatments (i.e., radiotherapy replanning secondary to bowel toxicity) with significant expected advantages in terms of treatment quality and overall clinical outcomes. Furthermore, the use of this monitoring approach may represent a key resource for coping strategies enhancement, especially in hospitals where a psychoncology service is not available.

As the volume of collected RWD increases, our next steps in the analysis side have to do with modeling the different scores and discovering a biomarker for the predictions of the QoL ones. Due to conflicting studies that do not allow to run multiple trials on the same cohort of patients, the enrollment has been slow, and therefore an amendment has been proposed to enlarge the cohort and grant the success of the trial. At the time being, patients affected by other pelvic cancer undergoing CRT are allowed in the study (e.g., endometrial, vaginal, vulvar, and rectal cancer). This expansion of the inclusion criteria is justified by the similarities of cervical to the other pelvic cancers in terms of treatment (there is a radiotherapy does overlap) and most importantly the common types of possible toxicity (linked to the irradiation of the same pelvic organs, i.e., bowel, bladder, rectum) that allow the reuse of the same questionnaires and eliminate differences in data analysis.

The limitation of the APACHE study lies with the automatic measurements. These need the compliance of the study participant, whose familiarity with activity trackers significantly declines with their age. The participant needs to understand the importance of the physiological data being collected and make sure their activity tracker is worn and is charged. Working with the unavoidable gaps in the collected physiological data is something we are investigating.

### Scoring Considerations

In section Study Description we presented the study design, which is complemented by the scoring mechanisms that we have been prepared prior to the launch of the study. The physiological scores combine diverse parameters, and their combination weights will be investigated to obtain meaningful results. All the other scores combine parameters that carry similar weights and hence there are three combination options:

A linear combination does not discriminate between number of events or their severity. As an example, consider four parameters with values 0–4, 4 indicating maximum severity. An average score of 1 is obtained with four 1's or three 0's and one 4. There are cases where the latter is more alarming than the former. Some of the questionnaires used in APACHE do have formal scoring suggestions (37–41), following the linear case.

Non-linear combination, on the other hand, allows the investigator to put more weight on either the number of events or their severity. Consider a set of possible events *x*_*n*_, each with a value of zero if there is no symptom and integer values larger than zero indicating increased severity of the symptom. The events are combined into a single score *s* using:


$$\begin{array}{l} s=\frac{1}{N}{\left(\overset{N}{\underset{n=1}{\sum }}x_{n}^{a}\right)}^{1/a} \end{array}$$

Selecting *a*>1 the investigator has a score that puts emphasis on event severity vs. event count. On the other hand, selecting *a* < 1 puts the emphasis on the event count. Selecting *a* = 1 leads to the linear case.

As already mentioned, the formal scoring of most of the questionnaires is linear. This is followed to facilitate clinical research. But to facilitate ML, we also experiment with non-linear scoring in APACHE. We are selecting the value of the exponent for each combination leading to the different scores, based on what the investigators need to emphasize with each one of them.

### Iterative Design Stage

The iterative design phase has recently started with an initial algorithm selection. Since there are 50 patients in the trial, there are 50 feature vector instances to train and evaluate the biomarkers. For this reason, algorithms like neural networks are not expected to be used. The biomarkers will most probably be based on a decision-tree classifier or random forests. Linear methods (with careful feature engineering) will be used as a baseline, together with their multiclass variants, since the decision boundaries are not expected to be linear.

The biomarkers will be trained using the leave-one-out method, each time keeping one patient for testing, training with 45 and using the remaining four for hyper-parameter tuning during validation. The performance will be reported as the number of correctly identified patients out of the 50 leave-one-out experiments.

### Early Findings and Next Steps

At the definition stage of the APACHE biomarkers discovery, the clinically significant outcomes that need to be predicted by the biomarker are selected. These are dictated by the goal of APACHE, that is, the QoL in terms of low toxicity adverse effects, as is quantified by the three scores of the lifestyle aspect. As APACHE trial starts producing RWD, we will be training our biomarker to predict significant variations of these scores. At some milestone of the trial (currently planned for its end on the 52nd week) the predictors of the biomarker will be able to determine if a significant improvement of the 3 scores associated with the QoL of the patients is to be expected.

Having trained the biomarker, we will be exploiting the explainable AI techniques described in section Composite Lifestyle Biomarker Discovery to determine the aspects in the life of the patient to coach in favor or against. This way, as discussed in section Using the Biomarkers for Digital Therapeutics, we will be driving DTx in this therapeutic area.

Although the RWD collection and scoring presented in this study is customized to the needs of the APACHE trial for cervical cancer, the methodology for capturing and combining data, and most importantly, that for discovering biomarkers, is applicable to other conditions as well. Preliminary results are available for the application of this methodology on RWD for obesity, where the discovered biomarker predicts significant short-term weight variations and general well-being, where the biomarker classifies the health outlook of general population, aiming at using it for risk assessment and the analysis of its decisions in virtual coaching. We have published these early results in Pnevmatikakis et al. (36), and our next steps in biomarker discovery research involve applying the discovery methodology in the APACHE data to predict the low-toxicity events.

## Conclusions

The APACHE study addresses a very important milestone, that is represented by the clinical validation of AI technologies when creating models based on PROMs capable of predicting any outcome of clinical value. In this modeling, endeavor the clinical validation still represents a bottleneck. In particular, the complexity of promoting RWE from basic clinical decision support (needs validation from an accountable “real doctor”) to a fully validated (rather “qualified”) digital biomarker RWD-from-lifestyle-raw-material based is quite significant. The challenge is represented by the robust regulatory framework set to qualify a classical biomarker, herein adopted to evaluate a digital one vs. strictly technological standards, requirements, and credentials. If we consider the privacy endeavor (related to innovative data capture and handling solutions) and the cyber-sec one, these alone represent entirely new dimensions entering the ethical/regulatory dialog.

In our perspective, extracting evidence with predictive values from lifestyle in a very homogeneous cohort (and a technological endeavor ethically and regulatory robust) of subjects undergoing state-of-the-art treatment magnifies its value by offsetting this RWE toward a very stable, and to a certain extent expected, clinical outcome progression (observational nature of the approach). In other words, a study like this creates the idea sandbox to evaluate the training of an ML algorithm in a low noise setting.

Clinically, extracting such RWE has significant implications. Lifestyle-driven and outcome-connected digital biomarkers with the predictive value could enrich the diagnostic tools with agile (and relatively inexpensive) indicators (easy to collect in a continuous fashion), for example, of the onset of significant toxicity from an oncological treatment.

Training cycle by training cycle, moreover, these digital biomarkers could pave the way to smart coaching that, in turn, could be promoted toward validated digital content as an active ingredient in a DTx perspective.

## Data Availability Statement

The clinical study APACHE and its raw data are property of Gemelli and cannot be shared.

## Ethics Statement

The studies involving human participants were reviewed and approved by Gemelli. The patients/participants provided their written informed consent to participate in this study.

## Author Contributions

SK, AP, and AC were the main editors. KK has contributed to the customization of Healthentia to support the APACHE trial. SK, AP, and AC have contributed to the ML of Healthentia. LB, VV, and GS have contributed to the study design and faciliating the APACHE trial. All authors contributed to the article and approved the submitted version.

## Conflict of Interest

SK, AP, AC, and KK were employed by company Innovation Sprint Sprl. The remaining authors declare that the research was conducted in the absence of any commercial or financial relationships that could be construed as a potential conflict of interest.

## Publisher's Note

All claims expressed in this article are solely those of the authors and do not necessarily represent those of their affiliated organizations, or those of the publisher, the editors and the reviewers. Any product that may be evaluated in this article, or claim that may be made by its manufacturer, is not guaranteed or endorsed by the publisher.
